# In Vitro Efficacy of Extracts and Isolated Bioactive Compounds from Ascomycota Fungi in the Treatment of Colorectal Cancer: A Systematic Review

**DOI:** 10.3390/ph16010022

**Published:** 2022-12-23

**Authors:** Cristina Luque, Ana Cepero, Gloria Perazzoli, Cristina Mesas, Francisco Quiñonero, Laura Cabeza, Jose Prados, Consolación Melguizo

**Affiliations:** 1 Institute of Biopathology and Regenerative Medicine (IBIMER), Center of Biomedical Research (CIBM), University of Granada, 18100 Granada, Spain; 2 Department of Anatomy and Embryology, Faculty of Medicine, University of Granada, 18071 Granada, Spain; 3 Instituto de Investigación Biosanitaria de Granada (ibs.GRANADA), 18014 Granada, Spain

**Keywords:** colorectal cancer, Ascomycota, bioactive compounds, functional extracts, fungi, antitumor activity, Eurotiales, Hypocreales, Pleosporales

## Abstract

Colorectal cancer (CRC) is the second leading cause of cancer-related deaths worldwide. Despite the advances and success of current treatments (e.g., chemotherapy), there are multiple serious side effects which require the development of new treatment strategies. In recent years, fungi have gained considerable attention as a source of extracts and bioactive compounds with antitumor capabilities because of their antimicrobial and antioxidant properties and even their anti-inflammatory and antiviral activities. In the present review, a systematic search of the existing literature in four electronic databases was carried out in which the antitumor activity against CRC cells of Ascomycota fungi extracts or compounds was tested. The systematical research in the four databases resulted in a total of 883 articles. After applying exclusion and inclusion criteria, a total of 75 articles were finally studied. The order Eurotiales was the most studied (46% of the articles), and the ethyl acetate extraction was the most used method (49% of the papers). *Penicillium* extracts and gliotoxin and acetylgliotoxin G bioactive compounds showed the highest cytotoxic activity. This review also focuses on the action mechanisms of the extracts and bioactive compounds of fungi against CRC, which were mediated by apoptosis induction and the arrest of the cell cycle, which induces a notable reduction in the CRC cell proliferation capacity, and by the reduction in cell migration that limits their ability to produce metastasis. Thus, the ability of fungi to induce the death of cancer cells through different mechanisms may be the basis for the development of new therapies that improve the current results, especially in the more advanced stages of the CCR.

## 1. Introduction

Colorectal cancer (CRC) is the third most common cancer type worldwide and the second deadliest malignancy for both sexes combined. In 2020, it was estimated that 935,000 deaths secondary to CRC occurred, and 1.9 million new cases were diagnosed. Specifically, the American Cancer Society had predicted that, in the United States in 2022, more than 100,000 people would be diagnosed with this type of cancer and approximately 52,000 deaths would be caused by CRC. It is known that CRC is associated with a high socioeconomic status, which explains its high incidence in European countries [1,2]. A clear correlation has been demonstrated between the development of the disease and environmental, hereditary, and lifestyle factors, including obesity, a sedentary lifestyle, smoking, processed or red meat, and alcohol. Nevertheless, certain preventive factors have also been identified, such as physical activity or a healthy diet (e.g., fruit, vegetables, fish, and garlic) [3].

The treatment of choice for CRC includes surgery (in the case of resectable tumours) and chemotherapy, such as 5-fluorouracil, oxaliplatin, irinotecan, and capecitabine, which can be used in mono-therapy or in combination with other drugs. However, chemotherapy causes multiple and serious secondary effects, such as high toxicity to healthy cells or cancer drug resistance. New biological drugs have been developed, such as monoclonal antibodies against epidermal growth factor receptor (EGFR) (cetuximab or panitumumab) or vascular endothelial growth factor (VEGF) (bevacizumab or ramucirumab), which also showed serious limitations both related to specific side effects (i.e., diarrhea, ocular-skin toxicity, etc) and their low efficacy in some types of CC [3,4]. Thus, it is necessary to develop new CRC therapeutic strategies [5,6]. Since the early ages, plants and fungi have aroused the interest of the scientific community as sources of bioactive compounds with antitumor capacities and, therefore, with a promising future as potential drugs [7,8,9].

In this context, fungi represent a relevant resource for isolating bioactive compounds, including polysaccharides, terpenes, terpenoids, proteins, amino acids, nucleosides, and phenols, among others, with antitumor activity and different mechanisms of action, from cell cycle arrest to the suppression of angiogenesis, and a metastatic capacity or induction of cell apoptosis [10]. In fact, Ascomycota and Basidiomycota are the two most researched fungal phyla in oncology [11,12]. Ascomycota is the largest fungal taxonomic group with around 6600 genera classified in approximately 130 orders [13]. Some of these genera, such as *Aspergillus*, *Penicillium*, *Fusarium,* or *Cordyceps*, are known to have antimicrobial and antioxidant properties and some additional characteristics, such as anti-inflammatory (*Cordyceps* and *Aspergillus*) or antiviral (*Penicillium*) characteristics. Likewise, many of the genera of the phylum Ascomycota showed significant activity against numerous types of tumors, such as leukemia, breast, lung, colon, or liver cancer [12,14,15,16]. Specifically, exopolysaccharides isolated from fungi, such as *Cordyceps sinensis* or *Ganoderma lucidum,* suppressed autophagolysosome formation in CRC culture cells. Furthermore, polysaccharides isolated from G. lucidum protected non-tumor colon cells from the accumulation of reactive oxygen species and potentiated the effects of 5-fluorouracil, decreasing tumor size and increasing survival in mouse models [17,18].

The aim of this systematic review was to analyze the most recent literature regarding the application of the fungal phylum Ascomycota for the CRC treatment using functional extracts or bioactive compounds with clearly identified antitumor activity, and gathers information on the processes through which they cause tumor cell death.

## 2. Results and Discussion

**Figure 1:**
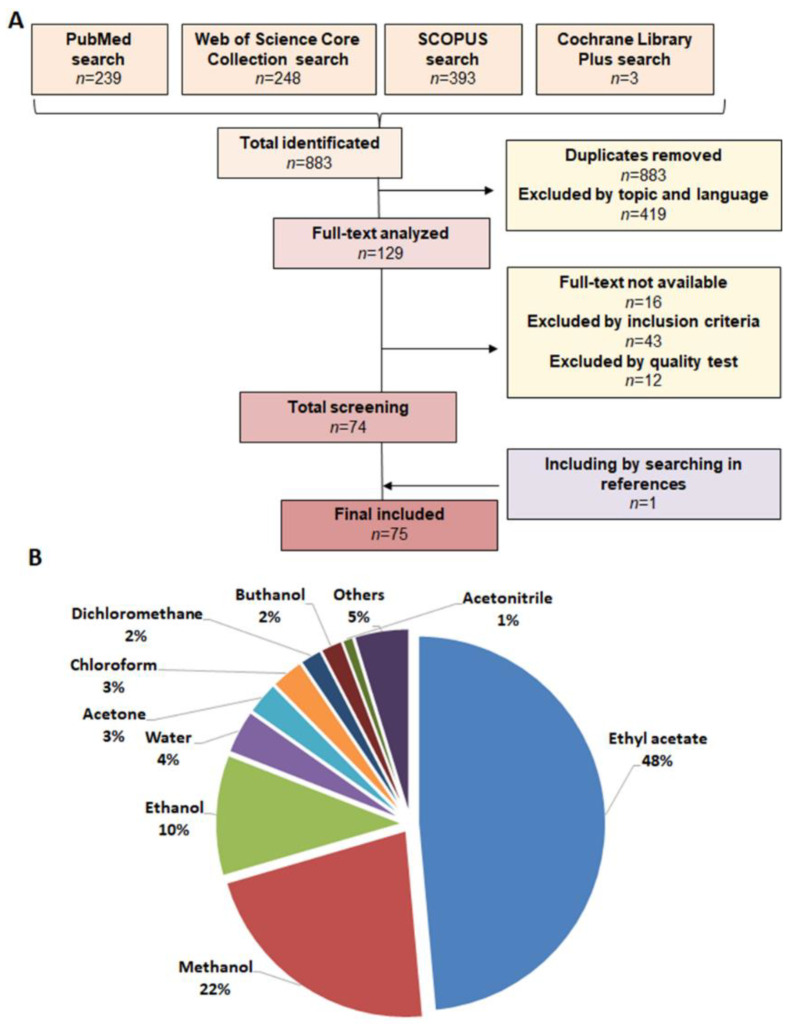
Flow diagram of the eligible studies included in this systematic review (**A**). Graphical representation of the method of extraction used to obtain the extract that was analyzed against tumor cells (**B**).

The systematic search in the four electronic databases resulted in a total of 883 articles (Figure 1A). After removing duplicates (*n* = 335) and articles that did not match the topic or language (*n* = 419), 129 articles were selected for full-text analysis, of which 16 were eliminated because the full text was not available. Thus, 113 articles were carefully analyzed and, after removing those that did not meet the inclusion criteria (*n* = 43) or did not comply with the minimum required quality (*n* = 12), 74 articles were finally obtained, to which one study was added by searching the references of the previous articles. Therefore, the present systematic review comprised a total of 75 articles. This systematic review analyzed a total of 13 orders of the phylum Ascomycota, and 41 articles studied different genera of the order Eurotiales (*Aspergillus*, *Penicillium*, *Byssochlamys*, *Talaromyces,* and *Neosartorya*). A total of eight genera of the order Hypocreales (*Beauveria*, *Bionectria*, *Cordyceps*, *Engyodontium*, *Fusarium*, *Metarhizium*, *Trichoderma,* and *Myrothecium*) were investigated in 16 articles. The genera of the orders Pleosporales (*Alternaria*, *Bipolaris*, *Curvularia*, *Drechslera*, *Paradendryphiella*, *Phoma,* and *Setophoma)* and Sordariales (*Chaetomium*, *Trichlocladium,* and *Scytalidium*) were studied in eight articles each. Other genera, such as *Pezizales, Capnodiales*, *Incertae sedis*, *Diaporthales,* or *Leotiales*, among others, were studied marginally. Finally, the most commonly used extraction method was ethyl acetate, followed by methanol, although many other approaches, such as methanol or water, were also applied (Figure 1B). Most publications assayed a functional extract or bioactive compound dissolved in DMSO against HCT-116 and HT-29, the most commonly used CRC cells lines. The MTT assay was the method of choice to determine antiproliferative activity on cells.

**Figure 2:**
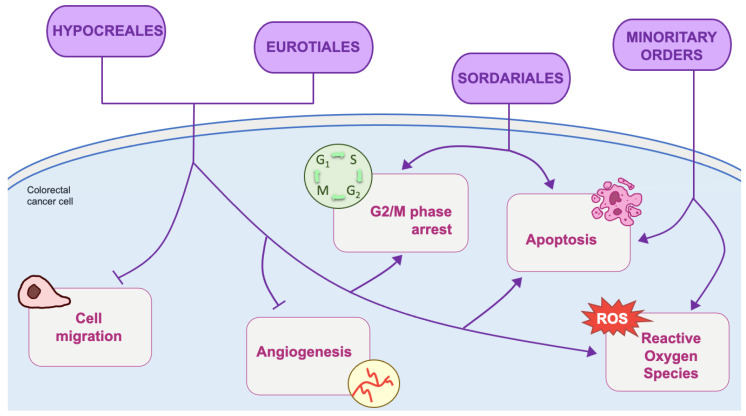
Mechanisms of action through which the different fungi of Ascomycota perform their effect as against CRC.

A total of 151 bioactive compounds have been isolated from the different orders of Ascomycota, tested in CRC cell lines (Supplementary Table S1), and analyzed to determine antitumor action mechanisms. As shown in Figure 2, the most studied pathways are those of the Eurotiales and Hypocreales orders.

### 2.1. Order Eurotiales

As shown in Table 1, 41 articles on the order Eurotiales were analyzed, showing that ethyl acetate was the most frequent extraction method (26 articles), followed by methanol [19,20,21] and ethanol [22,23,24]. Mohamed [25] employed sonication, centrifugation, and lyophilization. In addition, seven articles used differential extraction between a liquid medium and mycelia, most commonly employing ethyl acetate and methanol, respectively [26,27,28,29,30,31,32].

#### 2.1.1. Genus *Aspergillus*

Twenty-four articles analyzed species of the genus Aspergillus, of which eight articles tested functional extracts on CRC cell lines. Ethyl acetate extracts (five articles) showed IC_50_ values between 42.75 and 185.9 μg/mL [33,36,58]. Moreover, Ali et al. [36] reported that ethyl acetate extracts from nine different Aspergillus species induced death of 50.1 to 69.1% of HCT-116 cells. A similar extract obtained by Artasasta et al. [33] was reported to cause a significant reduction in the viability of WiDr cells. Asfour et al. [32] also used methanol as a mycelium extraction method, obtaining IC_50_ values between 15–100 μg/mL in HCT-116 cells, while Alasmary et al. [23] obtained an ethanolic extract with higher antitumor activity (IC_50_ 125 μg/mL) in the same cell line. Furthermore, Abd El-Hady et al. [19] tested a sequential extract of ethyl acetate, methanol, and dichloromethane (100 µg/mL) that induced significant cytotoxicity (15.8%) in the same cell line. Finally, a crude extract obtained by sonication, centrifugation, and lyophilization showed an IC_50_ value of 9.84 µg/mL in CaCo-2 cells [25]. Functional extract fractions (three articles) were tested on the HCT-116 cell line, with IC_50_ values between 5.28–193.64 µg/mL [24,33] and 15.8–88% cytotoxicity [19]. Interestingly, most of the extracts obtained from the genus Aspergillus were also tested on other cancer cell lines of liver, larynx, cervix, and breast [23,32,36,58], in which, in general, a higher cytotoxic effect was noted compared to CRC cells. Only the functional extracts obtained by Ashour et al. [24] reduced the IC_50_ to a greater extent in CRC (more than half the IC_50_) than in other tumor cells, such as those derived from liver and breast cancer.

On the other hand, 49 bioactive compounds from the genus Aspergillus were described in a total of 14 articles. For example, malformin C was effective in MC-38 and HCT-116 cell lines (IC_50_ 0.27 and 0.18 µM, respectively), with similar results being obtained in the murine pancreatic cancer cell line PanO2 and in the human lung adenocarcinoma cell line H1975. This bioactive compound induced G2/M phase arrest, DNA damage, apoptosis, autophagy, and necrosis [40]. Two of the most promising compounds in relation to the treatment of CRC were gliotoxin and acetylgliotoxin G, which showed very low IC_50_ values (0.41 and 1.06 µg/mL, respectively) against HCT-116 cells [44]. In fact, gliotoxin was also reported to have antitumor efficacy in chondrosarcoma, cervix, and glioblastoma cells [59,60]. In addition, asperphenin A showed greater activity in CRC cells than in breast cancer cells (IC_50_ 0.84 vs. 6.48 µM, respectively), inducing G2/M cell cycle arrest by the inhibition of tubulin polymerization, induction of apoptosis, and production of reactive oxygen species (ROS). In addition, asperphenin demonstrated a synergistic effect in combination with irinotecan and paclitaxel [20]. Other bioactive compounds, such as clavatustide B, inhibited the G1/S phase, while acetylaranotin, acetylapoaranotin, and deoxyapoaranotin induced apoptosis mediated by caspases 3, 9, and 8 [28,31,42]. Finally, isolated compounds from the genus aspergillus, such as asperphenin A, malformin C, or acetylapoaranotin have succeeded in taking a further step toward in vivo murine research, although more studies are needed [20,38,42].

#### 2.1.2. Genus *Penicillium*

The most relevant studies in the genus Penicillium used the ethyl acetate extraction method (five out of fourteen) [22,32] or methanol and ethanol methods (two out of fourteen) [49,54,56] to develop functional extracts that showed IC_50_ values between 0.2 and 102 µg/mL in CRC cells. Canturk et al. [56] and Dikmen et al. [38] showed that ethyl acetate extracts reduced the invasiveness of cancer cells by decreasing cell migration and the expression of genes related to angiogenesis and metastasis. In addition, a total of 48 bioactive compounds from different species of the genus Penicillium (nine of fourteen articles), including arenicolin A, isopenicin A, penipacids A, and norverrucosidin, were detected, showing the lowest IC_50_ values against HCT-116 (7.3 μg/mL), SW-180 (0.74 μg/mL), RKO (8.4 μg/mL), and HCT-116 cells (5.7 μg/mL), respectively. Furthermore, isopenicin A induced apoptosis and modulated proteins involved in cell cycle progression from G2 to M [21,29,30,52,53,55,57]. The anti-tumor activity of some of the extracts and bioactive compounds from the genus Penicillium were tested against breast, cervix, and liver cancer cells, obtaining similar results [21,32,52,53,55,57].

#### 2.1.3. Genera *Neosartorya, Byssochlamys* and *Talaromyces*

Only two studies on the genus Neosartorya developed ethyl acetate extracts (IC_50_ 139 µg/mL in HCT-116 cells) [45,47], while the other three articles reported the isolation of 18 bioactive compounds, such as gliotoxin and acetylgliotoxin, both of which are active against RKO cells (IC_50_ 1.24 μM). Moreover, reduced gliotoxin showed high toxicity in HCT-116 cells (IC_50_ 0.89 μM) [46,48], inducing apoptosis and ROS production [61]. On the other hand, the genera Talaromyces and Byssochlamys were studied by Castro-Carvalho et al. [45] and Khiralla et al. [39], respectively. The latter obtained an acetate extract with IC_50_ values of 56.3 and 30.4 µg/mL in HT-29 and HCT-116 CRC cell lines, respectively. Specifically, Byssochlamys extracts showed a significantly weaker antiproliferative effect on CRC cells compared to the breast cancer cell line MCF-7 (IC_50_ 1.51 µg/mL).

### 2.2. Order Hypocreales

#### 2.2.1. Genera *Cordyceps*, *Fusarium* and *Trichoderma*

As shown in Table 2, the studies on the order Hypocreales (16 articles) used a wide variety of extraction methods, although methanol and ethyl acetate were the most common. Four articles focused on the genus Cordyceps, obtaining methanol extracts (two articles) that showed IC_50_ values between 72.57 and 250 μg/mL against HCT-116, SW-480, and HCT-15, reducing both cell migration and cytoplasmic β-catenin [62,63]. An ethanol extract induced cell morphological changes and G2/M cell cycle arrest [64], and a butanol extract from Cordyceps militaris (sprouted soybean) induced a strong inhibition of HT-29 cell proliferation (56%) and G2/M phase arrest by blocking cyclin B1 and the expression of Cdc25c [65]. Lee et al. [64] tested this ethanol extract in a xenograft mouse model and found a significant inhibition of tumor growth and a reduction in mouse mortality. On the other hand, the genus Fusarium was studied in four articles, showing active functional extracts against CaCo-2, HCT-116, and HCT-8 cells (IC_50_ between 0.3779–98.68 μg/mL) [25,66,67,68]. In one article, standard camptothecin and camptothecin crude extract were isolated and tested against CaCo-2 cells, resulting in IC_50_ values of 2.41 and 0.291 μM, respectively [68]. This compound has been used for the development of a conjugate, CT-2106, that has been studied in a clinical trial in combination with 5-fluorouracil and folic acid (NCT00291785), whose results had not been reported yet. In addition, camptothecin is the precursor of irinotecan, an antitumor drug that, in combination with other anticancer agents, has been widely used in clinical trials and its clinical use is well accepted [69]. Finally, the genus Trichoderma was analyzed in four articles, obtaining functional extracts (IC_50_ between 11–100 μg/mL), fractions (IC_50_ between 7.3 and 14.9 μg/mL) [24,66,70], or bioactive compounds, such as trichodermaloid A and B (IC_50_ 9.3 and 8.6 μM in the SW-620 cell line, respectively) [71]. All of these findings are consistent with those obtained in other forms of tumors (breast, lung, liver, and cervix cancers, among others).

#### 2.2.2. Other Genera

The genera Beauveria, Bionectria, Engyodontium, Metarhizium, and Myrothecium were analyzed in one article each. 1-Hydroxy-10-methoxy-dibenz[b, e]oxepin-6,11-dione, chrysazin (IC_50_ > 30 μM), and globosuxanthone A (IC_50_ 10.7 μM) were isolated from the genus Beauveria and tested on HCT-15 cells [72]. Beauvericin, another compound from the genus Beauveria, has been used for in vivo assays in BALB/c and CB-17/SCID mice, decreasing tumor volumes and increasing necrotic areas of tumors, becoming a potentially interesting drug for the treatment of CRC [76]. Exopolysaccharides isolated from the genus Bionectria (0.45 mg/mL) significantly reduced HT-29 cell viability (15.42%) [73]. In addition, functional extracts from the genera Engyodontium and Myrothecium showed IC_50_ values of 2.5 μg/mL and 380 ng/mL in HCT-116 cells, respectively. Specifically, Myrothecium extract showed higher cytotoxic activity in breast MCF-7 cells (IC_50_ 107 ng/mL) and lower in the liver cell line HepG2 (IC_50_ 711 ng/mL) [49,73]. Finally, destruxins A, B, and E from the genus Metarhizium were tested in CaCo-2 and HCT-116 cells, showing IC_50_ values between 0.04 and 10 μM. However, they were also active against the KB-3-1 cell line derived from the epidermal carcinoma and A549 lung cancer cells. Furthermore, destruxin E induced ROS production and activated apoptotic caspases, even before mitochondrial membrane depolarization. The three destruxins reduced cell migration and angiogenesis, induced G0/G1 cell cycle arrest in the CaCo-2 cell line, and interfered with the MAPK and/or PI3K/Akt signaling pathways [74].

### 2.3. Orders Pleosporales and Sordariales

As shown in Table 3, the main studies of the order Pleosporales focused on the genus Alternaria (three out of eight articles) to obtain methanol extracts that were tested on HCT-116 and SW-480 cells (IC_50_ 5.39 and 12.37 μg/mL, respectively) [39,77]. In addition, the compound (6aR, 6bS, 7S)-3, 6a, 7, 10-tetra-hydroxy-4, 9-dioxo-4, 6a, 6b, 7, 8, 9-hexahydroperylene was isolated from the extract of the genus Alternaria with an IC_50_ value of 1.78 μmol/L in HCT-8 cells [78]. Pleosporales spp. were used to obtain an ethyl acetate extract that led to IC_50_ values of 69.4 μg/mL in HT-29 cells and 36.7 μg/mL in HCT-116 cells, while its cytotoxic activity in the MCF-7 breast cancer line was even half of that of the latter CRC line [39]. An aqueous extract and an organic residue obtained with dichloromethane were tested in HCT-116 cells, obtaining IC_50_ values between 12 and 100 μg/mL, respectively [66]. In addition, the genera Bipolaris, Phoma, Drechslera, and Curvularia were studied by obtaining functional extracts, which evidenced IC_50_ values ranging from 18.97 to 202.5 µg/mL against HCT-116, HT-29, and HCT-8 cells, with similar antiproliferative activity in breast cancer cell lines [23,36,70]. From the Drechslera genus, di-2-ethylhexyl phthalate was isolated (IC_50_ 9.5 ± 0.4 µg/mL in HCT-116 cell line). Another compound, (3R, 6R) hyalodendrin, was isolated from the genus Paradendryphiella (IC_50_ between 48.0 ± 9.3 nM and 163.7 ± 11.0 nM). Finally, seven bioactive compounds were isolated from the Setophoma genus and tested on SW-620 cells, with IC_50_ values between 0.21 (penicillixanthone A) and 19.12 μM (secalonic acid E) [23,79,80].

On the other hand, the order Sordariales (eight articles) included the study of the genus Chaetomium (six articles) (Table 4). The analysis of ethanol extracts against HCT-8, HCT-116, and HT-29 cells was reported with IC_50_ values ranging from 1.2 to 152.8 µg/mL [39,49,66] and twelve bioactive compounds. Specifically, Chaetocochins C and J resulted in the lowest IC_50_ values: 0.63 and 0.56 μM in SW-480 and HCT-116 cells, respectively [81,82,83]. Furthermore, Trichocladinols D-H, E, F, and G were isolated from the genus Trichlocladium (one article), showing IC_50_ values between 41.7 and 56.6 μM against the HCT-116 and SW-480 cells lines [84]. Finally, a total of 11 bioactive compounds were isolated from the genus Scytalidium in one study, including 5’-formyl-2’-hydroxyl-4’-methoxy-(E,E)-sorbophenone (IC_50_ 0.5 μM) and 5 ‘-formyl -2′-hydroxy-4′-methoxy-(E)-4-hexenophenone, which showed the best results (IC_50_ 2.5 μM) against SW-620 cells [85].

### 2.4. Minoritary Orders

As shown in Table 5, the antitumor activity of the order Capnodiales (three articles) was studied using the genera Cladosporium (functional extracts) and Zasmidium (bioactive compound). In fact, 8,8′-Bijuglone showed an IC_50_ value of 45 µg/mL in the HCT-116 cell line. Functional extracts and bioactive compounds from Cladosporium were tested on both CRC and breast cancer cells with a significant differential effect [39,86,87]. Taxol was one of the compounds, which, due to its potent antitumor effect, has not only been tested in several clinical trials but has also come to be used in clinics against CRC [88]. In addition, the genera Sclerotinia and Lachnum (order Helotiales) were processed to obtain the exopolysaccharide LEP-2b and derivates from the genus Lachnum, which showed high antitumor activity (e.g., IC_50_ of LEP-2b, 85.78 μg/mL) in the CT-26 cell line, among other tumor cells [89,90,91].

Studies on the order Diaportales (two articles) showed methanol and ethyl acetate extracts with IC_50_ values ranging from 5.63 to 24.47 µg/mL in SW-480 and HCT-116 cells lines [77], and the isolation of dicerandrol A and B with significant antitumor activity in HCT-116 CRC cells with IC_50_ values of 2.64 and 3.94 μM, respectively [92]. All of them were also highly effective against cell lines of other cancer types, such as breast, lung, and liver. The order Pezizales (two articles) was studied by Liu et al. [93] and Tang et al. [94]. The latter showed polysaccharides from the genus Morchella with high CRC cell cytotoxicity (IC_50_ between 1.229 and 2.827 mg/mL in CaCo-2 cells). This finding was supported by results in the hepatocellular cancer line HepG2. Similarly, four different compounds were isolated from the order Xylariales, highlighting 5-methylmellein and daldinone F, which showed significant antitumor activity (IC_50_ of 2 and 9.59 μM) in SW-480 and HCT-116 cells, respectively. Moreover, 5-Methylmellein showed activity against prostate and breast cancer cells. Indeed, it was encapsulated in nanoparticles, increasing the IC_50_ to <0.5 µg/mL, and inducing apoptosis, ROS production, and the loss of the mitochondrial membrane potential [95,96]. Finally, other orders, such as Boliniales, Incertae sedis, Leotiales, and Venturiales were studied using ethyl acetate extracts or bioactive compounds, such as xylarenone D, greensporone C, and O-desmethyl greensporone C, which were effective against CRC cells (IC_50_ 1.5, 7.5 and 13.8 μM, respectively), among other cancer types (melanoma, glioblastoma, and leukemia) [34,39,49,97].

**Table 5:** Antitumor activity of the extracts or isolated compounds from Minoritary orders in CRC cancer cell lines.

Order	Genus	Isolated from	Extraction	Isolated Compounds	Cell Line/Administration/Cytotoxicity Assay	Compound and IC_50_ or Cell Death (%)	Mechanism of Action	Reference
Capnodiales	*Zasmidium*	Foliage	Ethyl acetate (EtOAc)	8,8′-Bijuglone	HCT-116 DMSO MTT	8,8′-Bijuglone: 45 μg/mL	-	[87]
Capnodiales Incertae sedis	*Cladosporium* *Hansfordia*	*C. procera* *Vernonia amygdalina*	EtOAc	-	HT-29 and HCT-116 - MTT	*C. cladosporioides* extracts 1 (HT-29: 77.7 μg/mL; HCT-116: 45.6 μg/mL), 2 (HT-29 and HCT-116: >100 μg/mL); *H. sinuosae* extract: HT-29 (47.6 μg/mL), HCT-116 (>100 μg/mL)	-	[39]
Capnodiales	*Cladosporium*	Medicinal plants	Sodium bicarbonate	Taxol	HCT-15 Methanol MTT	Taxol: 3.5 μM	-	[86]
Helotiales	*Sclerotinia*	Contaminated soybean seed	Ethanol	-	HCT-8 - MTT	Fractions ethyl acetate (48.03 μg/mL), F3 (250.50 μg/mL)	-	[91]
Helotiales	*Sclerotinia*	Soybeans	Trituration and boiled	-	CCD-18Co and HT-29 Alone Electronic counter	Aqueous extract: CCD-18Co (11%), HT-29 (58%)	Aqueous extract induces ROS and extrinsic pathway	[90]
Helotiales	*Lachnum*	-	Ethanol	Exopolysaccharide LEP-2b	CT-26 DMSO MTT	LEP-2b: 8816.27 μg/mL; PLEP-2b: 85.78 μg/mL; SLEP_2b: 154.52 μg/mL	-	[89]
Diaporthales	*Phomopsis*	*Acanthus ilicifolius*	Methanol	Phomolactonexanthone A (1), B (2) and C (3) Dicerandrol A (4), B (5) and C (6) Deacetylphomoxanthone B (7) Penexanthone A (8)	HCT-116 Alone MTT	(1) and (2): >50 μM; (3): 44.06 μM; (4): 2.64 μM; (5): 3.94 μM; (6): 42.63 μM; (7): 7.12 μM; (8): 6.92 μM	-	[92]
Diaporthales	*Phomopsis*	*Miquelia dentata*	Methanol EtOAc	-	SW-480 and HCT-116 - Hoechst 33342	Methanol extract: HCT-116 (24.47 μg/mL), SW-480 (14.45 μg/mL) Ethyl acetate extract: HCT-116 (5.63 μg/mL), SW-480 (23.5 μg/mL)	-	[77]
Pezizales	*Morchella*	-	Hot water	Polysaccharides	CaCo-2 Alone Methylene Blue Assay	PMEP: 1.840 mg/mL; Ac-PMEP 1 (2.094 mg/mL), 2 (2.827 mg/mL), 3 (1.229 mg/mL)	-	[94]
Pezizales	*Morchella*	-	Pulsed electric field	-	HT-29 Alone MTT	M2 fraction: 54.29%	M2 fraction induces apoptosis	[93]
Xylariales	*Xylaria*	*Aegle marmelos*	EtOAc	5-methylmellein	HCT-116 Alone SRB	5-methylmellein: 2.0 μg/mL; 5-methylmellein nanoparticle: <0.5 μg/mL	5-methylmellein nanoparticle induces apoptosis, ROS and mitochondrial membrane potential loss	[95]
Xylariales	*Daldinia*	*Tenodera aridifolia*	EtOAc	Daldinone F (1) Nodulisporin G (2) Dalmanol C (3)	SW-480 and HCT-116 Alone MTT	(1): SW-480 (9.59 μM), HCT-116 (>20 μM); (2) and (3): SW-480 and HCT-116 (>20 μM)	-	[96]
Boliniales	*Camarops*	*Alibertia macrophylla*	EtOAc	Xylarenone C and D	HCT-8 Alone MTT	Xylarenones C (1.9 μg/mL), D (1.5 μg/mL)	Xylarenone D shows weak AChE inhibitory activity	[34]
Leotiales	*Halenospora*	Wood		Greensporone A (1), C (2) Dechlorogreensporone A (3), D (4) *O*-Desmethylgreensporone C (5)	HT-29 DMSO CellTiter 96 Aqueous One Solution Cell Proliferation Assay	(1) and (3): >20 μM; (2): 7.5 μM; (4): 25.4 μM; (5): 13.8 μM	-	[97]
Venturiales	*Ochroconis*	-	EtOAc	-	HCT-116 DMSO SRB	*Ochroconis* sp. extract: 70.5 μg/mL	-	[49]

PMEP: polysaccharides extracted from Morchella angusticepes Peck; Ac-PMEP: acetylated derivatives of PMEP; SRB: sulforhodamine B; MTT: 3-(4,5-dimethytlthiazol-2-yl)-2,5-diphenyltetrazolium bromide; IC_50_: half maximal inhibitory concentration; DMSO: dimethyl sulfoxide; ROS: reactive oxygen species; AChE: acetylcholinesterase; PLEP: phosphorylated polysaccharide; SLEP: sulfated polysaccharide.

## 3. Materials and Methods

A complete method was thoroughly organized with the collection of data and the steps of analysis, including the protocol registration (https://doi.org/10.17605/OSF.IO/X5KTD accessed on 10 November 2022).

### 3.1. Study Eligibility

Since the purpose of this review was to compile the most recent and representative knowledge of the antitumor capacities against colorectal cancer of bioactive compounds isolated from Ascomycota fungi or their functional extracts, a bibliometric analysis was carried out. A period of 10 years was established, considering older results obsolete (Burton–Kebler index for obsolescence) and including more than half of the actual disponible papers [98].

### 3.2. Inclusion Criteria

Research articles published in English from January 2011 to October 2021 in which extracts, or compounds isolated from Ascomycota fungi, had their antitumor activity on CRC cell lines tested were included in this systematic review. The research articles had been published in peer reviewed journals with the full text accessible.

### 3.3. Exclusion Criteria

Papers in which any colon cancer cell line was not used, or the bioactive compound or extract tested had no antitumor activity, were excluded. Furthermore, publications in which the bioactive compound was synthesized/purchased, or the extraction methodology was not specified, were also excluded. Finally, studies that did not exceed the minimum requirements of an in vitro study or with a low quality of the study, were excluded from the present review.

### 3.4. Data Sources

Four electronic databases were used to perform the systematic review: MedLars Online International Literature, via PubMed, SCOPUS, Web of Science Core Collection, and the Cochrane Library Plus. Firstly, the following Medical Subject Headings (MeSH) were defined to use as descriptors in Pubmed: “Colorectal Neoplasms”, “Fungi”, “Ascomycota”, and “Aspergillus”. The final equation was ((“Colorectal Neoplasms”[MeSH Terms] OR ((“colon”[Title/Abstract] OR “rectal”[Title/Abstract] OR “colorectal”[Title/Abstract] OR “colonic”[Title/Abstract]) AND (“cancer*”[Title/Abstract] OR “tumor*”[Title/Abstract] OR “tumour*”[Title/Abstract] OR “neoplasm*”[Title/Abstract] OR “carcinoma*”[Title/Abstract] OR “adenocarcinoma*”[Title/Abstract]))) AND (“Fungi”[MeSH Terms] OR “fung*”[Title/Abstract] OR “Ascomycota”[MeSH Terms] OR “Ascomycota”[Title/Abstract] OR “Ascomycetes”[Title/Abstract] OR “Aspergillus”[MeSH Terms] OR “Aspergillus”[Title/Abstract] OR “Chaetomium”[Title/Abstract] OR “Cordyceps”[Title/Abstract] OR “Neosartorya”[Title/Abstract]) AND (“Metabolite”[Title/Abstract] OR “extract*”[Title/Abstract] OR “bioactive compound*”[Title/Abstract])). The equation was adapted for the other three databases. The final list of studies included was completed by a manual search from the references of the selected publications.

### 3.5. Study Selection

Two of the authors (C.L. and A.C.) performed the bibliographic search, screened the abstract of the resulting publications, and selected the adequate ones for a fully-text review. Editorials, conference papers, bibliographic and meta-analysis reviews, book chapters, epidemiological studies, and case reports were excluded. In the following stage of the selection process, the same authors analyzed and included or excluded the full-text articles. Because the aim of this study was to review the current data available relating to in vitro publications, in vivo and clinical trials were manually excluded.

### 3.6. Data Extraction

Once the list of the articles included in the study was obtained, the same authors independently evaluated and extracted data from the selected studies according to the Cohen kappa statistical test for agreements (more than 0.8) [99]. Any discrepancy was resolved by a consensus between C.L. and A.C. or two more authors (J.P and C.M), if necessary. All of the selected articles were analyzed for quality using a specific questionnaire for in vitro studies with a first part in which the minimum requirements of an in vitro study were determined (score > 6), and a second part in which the quality of the study was analyzed (0–6 = low; 7–14 = good; 15–20 = excellent) based on materials and methods, results, and conclusions. Publications were classified according to the order of the studied fungi and the extracted data are condensed in Table 1, Table 2, Table 3, Table 4 and Table 5. To facilitate the interpretation of the selected studies, reference genera of fungi studied, where the fungi were isolated from, the extraction method, isolated compounds, the cell line used, cytotoxicity activity, and the mechanism of action.

## 4. Conclusions

This systematic review focused on in vitro studies on the antitumor activity of extracts and compounds isolated from fungi of the phylum Ascomycota. Of all of the genera analyzed in the literature, *Penicillium*, *Fusarium,* and *Chaetomium* produced the extracts with the greatest antitumor activity in CRC. A wide variety of bioactive compounds have been isolated from different genera of the phylum, some of which are particularly interesting given their high anticancer activity against this tumor. Although current results are very promising, more research is needed on genera that have been less studied. It is also important to move towards in vivo studies and/or clinical trials of the extracts and/or bioactive compounds with the aim that they could be used as a therapy against CRC in the future.

## Figures and Tables

**Table 1:** Antitumor activity of the extracts or isolated compounds from Eurotiales order in CRC cancer cell lines.

Genus	Isolated from	Extraction	Isolated Compounds	Cell Line/Administration/Cytotoxicity Assay	Compound and IC_50_ or Cell Death (%)	Mechanism of Action	Reference
*Aspergillus*	Submerged decaying wood	Methanol/ Dichloromethane	Asperphenins A (Asp. A) Asperphenins B (Asp. B)	RKO Alone and in combination with irinotecan and TXL SRB	Asp. A: 0.84 µM Asp. B: 1.26 µM Asp. A + Irinotecan at 1.25, 2.5, 5 and 10 µM (0.652, 0.811, 0.756, 0.694 and 0.652 μg/mL, respectively) Asp. A + TXL at 0.16, 0.8, 4 and 20µM (1.673, 1.925, 0.742 and 1.185 μg/mL, respectively)	Asp. A inhibits tubulin polymerization, generates ROS and induce G2/M arrest and apoptosis Asp. A and Irinotecan show synergism	[20]
*Aspergillus*	*Neopetrosia chaliniformis*	Ethyl acetate (EtOAc)	-	HCT-116 - MTT	Fractions I (193.64 μg/mL), II (5.28 μg/mL), III (15.82 μg/mL), IV (10.27 μg/mL), V (45.57 μg/mL)	-	[33]
*Aspergillus* *Penicillium*	*Stylissa carteri* Sediment *Hyrtios erectus* Marine sediment	Liquid medium: EtOAc Mycelia: Methanol	-	HCT-116 DMSO SRB	Penicillium Mycelia (M): between 15.00 and 92.60 μg/mL; Broth (B): between 74.20 and >100 μg/mL Aspergillus M: between 48.00 and 97.00 μg/mL; B: between 42.75 and 90.00 μg/mL	-	[32]
*Aspergillus*	*Bruguiera gymnorrhyza*	EtOAc	-	HCT-116 Alone MTT	EtOAc extract (EAE): 10.1 μM	-	[15]
*Aspergillus*	*Achillea fragrantissima*	Ethanol	-	HCT-116 DMSO MTT	Subfractions N. Hexane (76 μg/mL), EtOAc (26.3 μg/mL), Butanol (89.1 μg/mL)	-	[24]
*Aspergillus*	Marine Alga	EtOAc	Allianthrone A-C	HCT-116 DMSO MTT	Allianthrone A, B and C: >20 μM	-	[34]
*Aspergillus*	-	Liquid medium: Methanol Mycelia: EtOAc	Aspergiside B (1) Aspergisidone (2) Emeguisin A (3) Folipastatin (4) Aspergillusidone C (5) Unguinol (6) 2-Chlorounguinol (7) 2,4-Dichlorounguinol (8) Nidulin (9)	HCT-116 - MTT	(1): 3.98%; (2): 20.19%; (3): 23.5 µM; (4): 53.69%; (5): 7.84%; (6): 21.59%; (7): 4.92%; (8): 52.49%; (9): 3.58%	-	[31]
*Aspergillus*	Sponge	EtOAc	Violaceimide A-E	HCT-8 - MTT	Violaceimides A (1.5 μM), B (2.51 μM), C, D (>20 μM), E (>100 μM)	-	[35]
*Aspergillus*	*Sinularia* sp.	EtOAc Methanol Dichloromethane	-	HCT-116 DMSO MTT	CH_2_Cl_2_ extract: 15.8% Fractions 1 (29%), 2 (38%), 2c (88%), 2d (85%)	-	[19]
*Aspergillus*	Soil	EtOAc	-	HCT-116 DMSO SRB	*A. niger* (69.1%), *A. nomius* (68.2%), *A. terreus* (63.7%), *A. fumigatus* (60.3%), *A. flavus* (55.7%), *A. candidus* (55.1%), *A. stellifer* (51.9%), *A. oryzae* (50.7%), *A. violaceus* (50.1%)	-	[36]
*Aspergillus*	*Neopetrosia chaliniformis*	EtOAc	-	WiDr - MTT	EAE of NC01 (87.89%), NC02 (102.43%), NC03 (70.98%), *A. nomius* (29.69%), NC07 (85.96%), NC08 (4.48%), NC09 (53.96%)	EAE of NC01 induces apoptosis	[37]
*Aspergillus*	Lake	EtOAc	-	CaCo-2 DMSO WST-1 and RTCA	EAE: 185.9 μg/mL	-	[38]
*Aspergillus* *Byssochlamys*	*Calotropis procera* *Catharanthus roseus* *Euphorbia prostrata* *Vernonia amygdalina*	EtOAc	-	HT-29 and HCT-116 - MTT	*A. terreus* 1: HT-29 and HCT-116: >100 μg/mL *A. terreus* 2: HT-29: >100 μg/mL, HCT-116: 30.7 μg/mL *Byssochlamys*: HT-29: 56.3 μg/mL, HCT-116: 30.4 μg/mL	-	[39]
*Aspergillus*	Sand soil	N-butyl alcohol	Malformin C	MC-38 and HCT-116 - Methylene blue	Malformin C: 0.27 and 0.18 µM (MC-38 and HCT-116, respectively)	Malformin C induces G2/M arrest, DNA damage, apoptosis, autophapy and necrosis	[40]
*Aspergillus*	*Xenograpsus testudinatus*	Liquid medium: EtOAc Mycelia: Methanol	Clavatustide B	SW-480 - CCK-8	Clavatustide B: 37%	Clavatustide B inhibits G1/S phase cell cycle transit	[28]
*Aspergillus*	Solar saltern	Liquid medium: EtOAc Mycelia: Ethanol	Ergosterol (1) Rosellichalasin (2) Cytochalasin E (3)	RKO DMSO MTT	(1): 3.3 μM; (2): 62.3 μM; (3): 37.3 μM	-	[27]
*Aspergillus*	*Malus halliana*	EtOAc	Asperterone B and C	SW-1116 - MTT	Asperterones B (57.5 µM), C (1.0 µM)	-	[41]
*Aspergillus*	*Eudistoma vannamei*	Liquid medium: EtOAc Mycelia: Methanol	Isocoumarin (R)-mellein (1) Penicillic acid (2) *cis*-4-hydroxymellein (3) *trans*-4-hydroxymellein (4)	HCT-8 DMSO MTT	(1), (3) and (4): >25 μg/mL; (2): 8.76 μg/ml	-	[26]
*Aspergillus*	Marine sediment	EtOAc	Acetylaranotin (1) Acetylapoaranotin (2) Deoxyapoaranotin (3)	HCT-116 - MTT	(1): 21.2 μmol/L; (2): 13.8 μmol/L; (3): 52 μmol/L	All compounds induce caspases 3-, 9- and 8-dependent apoptosis	[42]
*Dichotomomyces* *(Aspergillus)*	*Lobophytum crassum*	EtOAc	Pityriacitrin	HCT-116 Alone SRB	Pityriacitrin: 35.1 µM	-	[43]
*Dichotomomyces* *(Aspergillus)*	Marine sediment	EtOAc	Bis(dethio)bis(methylsulfanyl)gliotoxin (1) 6-acetylbis(dethio)bis(methylsulfanyl)gliotoxin (2) Acetylgliotoxin G (3) Gliotoxin (4) Acetylgliotoxin (5) Fiscalin B (6)	HCT-116 DMSO MTT	(1): 23.56 μg/mL; (2): 35.97 μg/mL; (3): 1.06 μg/mL; (4): 0.41 μg/mL; (5): >50 μg/mL; (6): 33.51 μg/mL	-	[44]
*Emericella* *Aspergillus)*	Soil	Sonication, centrifugation and lyophilization	-	CaCo-2 - MTT	Crude extract: 9.84 μg/mL	-	[25]
*Eurotium* *(Aspergillus)*	-	Ethanol	1,8-Dihydroxy-3-methoxy-6-methyl- anthraquinone	HCT-116 DMSO MTT	*Eurotium* extract: 125.0 μg/mL 1,8-Dihydroxy-3-methoxy-6-methyl- anthraquinone: 18.6 μg/mL	-	[23]
*Neosartorya* *Aspergillus* *Talaromyces*	*Aka coralliphaga* *Porites lutea* Coastal forest soil *Rhabdermia* sp. *Chondrilla australiensis* *Clathria reianwardii*	EtOAc	-	HCT-116 and HT29 Alone or combined with Dox MTT	-	-	[45]
*Neosartorya*	*Rumphella* sp.	EtOAc	Chevalone C (1) Nortryptoquivaline (2) Tryptoquivaline H (3) Fiscalin A (4) *epi*-fiscalin A (5) and C (6) *epi*-neofiscalin A (7)	HCT-116 DMSO MTT	(1): 153 μM; (2): 114 μM; (3): 202 μM; (4): 123 μM; (5): 277 μM, (6): 86 μM; (7): 203 μM	-	[46]
*Neosartorya*	*Aka coralliphaga* Coastal forest soil *Porites lutea*	EtOAc	-	HCT-116 and HT-29 DMSO MTT	Extracts 1 (HCT-116 and HT-29: >200 μg/mL), 2 (HCT-116: 139 μg/mL, HT-29: 200 μg/mL), 3 (HCT-116: 189 μg/mL, HT29: 196 μg/mL)	-	[47]
*Neosartorya*	*Acanthaster planci*	EtOAc	1,2,3,4-Tetrahydro-2-methyl-3-methylene-1,4-dioxopyrazino [1,2-a]índole (1) 1,2,3,4-Tetrahydro-2-methyl-1,3,4-trioxopyrazino [1,2-a]índole (2) Gliotoxin (3) Acetylgliotoxin (4) Reduced gliotoxin (5) 6-Acetylbis(methylthio)gliotoxin (6) Bisdethiobis(methylthio)gliotoxin (7) Didehydrobisdethiobis(methylthio)gliotoxin (8) Bis-*N*-norgliovictin (9)	HCT-116 and RKO DMSO MTS	(1): HCT-116: 10.34 μM, RKO: 33.56 μM; (2), (6), (8) and (9): HCT-116 and RKO: >50 μM; (3) and (4): HCT-116: 1.24 μM, RKO: 0.80 μM; (5): HCT-116: 0.89 μM, RKO: 1.24 μM; (7): HCT-116: 8.59 μM, RKO: 10.32 μM	-	[48]
*Penicillium*	*Sonneratia* sp.	EtOAc	-	HCT-116 DMSO SRB	*Eupenicillium* sp.: 13.9 μg/mL *P. decumbens*: 0.2 μg/mL	-	[49]
*Penicillium*	*Isodon*	EtOAc	Isopenicin A	SW-480 DMSO MTT	Isopenicin A: 8.33 μmol/L	Isopenicin A regulates cycle progression from G2 to M and induces apoptosis	[50]
*Penicillium*	-	Methanol	Arenicolin A	HCT-116 - CellTiter-Glo	Arenicolin A: 7.3 μM	-	[21]
*Penicillium*	*Anemonia sulcata*	EtOAc	*cis*-bis(methylthio)silvatin	CaCo-2 and HCT-116 DMSO MTT	*cis*-bis(methylthio)silvatin: HCT-116 29.29 µM, CaCo-2 35.31 µM	-	[51]
*Penicillium*	Marine water	-	Exopolysaccharides	CaCo-2 - SRB	Exopolysaccharides: 3.21 mg/mL	-	[52]
*Penicillium*	*Trichocolea tomentella*	EtOAc	Epoxydon (1) 3,6,8-trihydroxy-1-methylxanthone (2) Gentisyl alcohol (3) (*R,S*)-1-phenyl-1,2-ethanediol (4) Dehydrodechlorogriseofulvin (5) Dechlorogriseofulvin (6) Griseofulvin (7) Ethylene glycol benzoate (8) Alternariol (9) Griseoxanthone C (10) Drimiopsin H (11) Griseophenone B (12) and C (13)	HT-29 - -	(1): 14.1 μM; (2), (4), (5), (6), (7), (8), (9), (10), (11), (12) and (13): >20 μM; (3): 6.4 μM	-	[53]
*Penicillium*	Lake	EtOAc	-	CaCo-2 DMSO WST-1 and RTCA-DP	EAE: 55.2 μg/mL	EAE decreases angiogenesis and metastasis gene expression, cell migration and invasively	[54]
*Penicillium*	Plant leaf	EtOAc	Kongiiline A (1), B (2) Pebrolide (3) 1-deoxypebrolide (4) Asperphenamate (5) Asperphenamate B (6), C (7) N-benzoyl-phenylalaninol (8) Orsellinic acid (9) Mycophenolic acid (10) 5,7-dihydroxy-4-methylphthalide (11)	HCT-116 DMSO MTT	(1), (2), (3), (4), (8), (9) and (11): 100 μM; (5): 88.16 μM; (6): 77.68 μM; (7): 91.72 μM; (10): 36.92 μM	-	[55]
*Penicillium*	Marine sediment	Liquid medium: Methanol Mycelia: 80% acetone/ H_2_O	(–)-Brevianamide C	HCT-116 DMSO SRB	(–)-Brevianamide C: 15.6 µM	-	[29]
*Penicillium*	*Isurus oxyrinchus*	Liquid medium: EtOAc Mycelia: Methanol	Fructigenine A (1) Verrucosidin (2) Norverrucosidin (3)	HCT-116 - Crystal violet	(1): 40.5 μg/mL; (2): 30.8 μg/mL; (3): 5.7 μg/ml	-	[30]
*Penicillium*	-	EtOAc	-	CaCo-2 DMSO WST-1 and RTCA-DP	EAE: 102 μg/mL	EAE induces apoptosis	[56]
*Penicillium*	Marine sediment	Acetone	Penipacids A and E	RKO - MTT	Penipacids A (8.4 μM), E (9.7 μM)	-	[57]
*Penicillium*	*Terminalia chebula* Retz	EtOAc Ethanol	-	CaCo-2 DMSO MTT	EAE of IR-4 (55 μg/mL), IR-6 (44 μg/mL), IR-7 (67 μg/mL) Ethanol extract of IR-6: 71 μg/mL	-	[22]

SRB: sulforhodamine B; ROS: reactive oxygen species; TXL: paclitaxel; CH_2_Cl_2_ extract: sequential ethyl acetate, methanol, and dichloromethane extract; Dox: doxorubicin; CCK8: cell counting kit-8; WST-1: 4-[3-(4-Iodo-phenyl)-2-(4-nitrophenyl)-2H-5 tetrazolio]-1,3-benzene disulphonate; RTCA-DP: real-time cell analysis system; EAE: ethyl acetate extract; MTS: ((3-(4,5-dimethylthiazol-2-yl)-5-(3-carboxymethoxyphenyl)-2-(4-sulfophenyl)-2H-tetrazolium)) assay; mRNA: messenger RNA; MTT: 3-(4,5-dimethytlthiazol-2-yl)-2,5-diphenyltetrazolium bromide; IC_50_: half maximal inhibitory concentration; DMSO: dimethyl sulfoxide.

**Table 2:** Antitumor activity of the extracts or isolated compounds from Hypocreales order in CRC cancer cell lines.

Genus	Isolated from	Extraction	Isolated Compounds	Cell Line/Administration/Cytotoxicity Assay	Compound and IC_50_ or Cell Death (%)	Mechanism of Action	Reference
*Beauveria*	Marine sponge	Acetone	1-Hydroxy-10-methoxy-dibenz[*b, e*]oxepin-6,11-dione (1) Chrysazin (2) Globosuxanthone A (3)	HCT-15 - MTT	(1) and (2): >30 μM; (3): 10.7 μM	-	[72]
*Bionectria*	*Psidium guajava*	Ethanol	Exopolysaccharides	HT-29 Alone MTT	Exopolysaccharides (84.58%)	-	[73]
*Cordyceps*	-	Methanol	-	HCT-116 and SW-480 DMSO MTT	Extract: HCT-116: >250 μg/mL; SW-480: 178.70 μg/mL	Extract reduces cell migration and cytoplasmic β-catenin	[63]
*Cordyceps*	-	Ethanol	-	RKO Distilled water CCK-8	-	Extract induces cell morphological changes, G2/M phase arrest and apoptosis	[64]
*Cordyceps*	-	Methanol:water (80:20 *v*/*v*)	-	HCT-15 - SRB	Extract: 72.57 μg/mL	-	[62]
*Cordyceps*	-	Methanol Buthanol	-	HT-29 Alone EZ-CyTox kit	GSC (46.56%) BuOH extract of *C. militaris* (36.23%)	GSC induces cell morphological changes and G2/M phase arrest, blocking the cyclin B1 and Cdc25c protein	[65]
*Engyodontium*	*Terminalia* sp.	Ethyl acetate (EtOAc)	-	HCT-116 DMSO SRB	EtOAc extract (EAE): 2.5 μg/mL	-	[49]
*Fusarium*	Soil	EtOAc	-	HCT-8 and HCT-116 DMSO CellTiter-Glo assay	EAE: HCT-8: 0.3779 μg/mL, HCT-116: 15.86 μg/mL	-	[67]
*Fusarium*	-	-	Camptothecin	CaCo-2 DMSO:Methanol (1:50) Alamar blue assay	Standard camptothecin: 2.41 μM; Crude camptothecin extract: 0.591 μM	-	[68]
*Fusarium*	Soil	Sonication, centrifugation and lyophilization	-	CaCo-2 - MTT	Crude extract: 6.24 μg/mL	-	[25]
*Metarhizium*	-	Liquid medium: Filtration Acetonitrile and sodium chloride	Destruxin A (1), B (2) and E (3)	CaCo-2 and HCT-116 DMSO MTT	(1): CaCo-2 (2.18 μM), HCT-116 (2.06 μM); (2): CaCo-2 (1.34 μM), HCT-116 (3.22 μM); (3): CaCo-2 (0.05 μM), HCT-116 (0.04 μM)	(3) activates apoptotic caspases and induces ROS All destruxins induce G0/G1 phase arrest in CaCo-2 cells, reduce cell migration, have antiangiogenic activity and interfered with the MAPK and/or PI3K/Akt signaling pathways	[74]
*Myrothecium*	*Calotropis procera*	Liquid medium: Chloroform and methanol (2:1 *v*/*v*)	-	HCT-116 DMSO Crystal violet staining	Extract: 380 ng/mL	-	[75]
*Trichoderma*	*Dysidea* sp.	EtOAc	Trichodermaloid A (1), B (2) and C (3) Aspergilloid G (4) Rhinomilisin E (5) and G (6)	SW-620 DMSO MTT	(1): 9.3 μM; (2): 8.6 μM; (3): 12.7 μM; (4) and (6): >32 μM; (5): 22.7 μM	-	[71]
*Trichoderma*	*Polygonum aviculare*	Ethanol	-	HCT-116 DMSO MTT	Fractions F2 (14.9 μg/mL), F4 (7.3 μg/mL), F5 (7.61 μg/mL)	-	[24]
*Trichoderma*	*Stylissa* *flabelliformis*	EtOAc	-	WiDr - MTT	EAE: 88.88 μg/mL	EAE induces apoptosis	[70]
*Trichoderma* *Fusarium*	*Bacopa monnieri*	Methanol	-	HCT-116 DMSO MTT	B1, B20, BX1: O and A (>100 μg/mL); *T. aureoviride*: O (11 μg/mL), A (>100 μg/mL); *Fusarium* sp. 6241: 5 μg/mL; *F. oxysporum*: O (22 μg/mL), A (98.68 μg/mL)	-	[66]

CCK8: cell counting kit-8; BuOH: buthanol extract; GSC: cordyceps militaris cultivated on germinated soybeans; SRB: sulforhodamine B; MTT: 3-(4,5-dimethytlthiazol-2-yl)-2,5-diphenyltetrazolium bromide; IC_50_: half maximal inhibitory concentration; DMSO: dimethyl sulfoxide; ROS: reactive oxygen species; O: organic residue; A: water extract; EAE: ethyl acetate extract; EE: ethanol extract.

**Table 3:** Antitumor activity of the extracts or isolated compounds from Pleosporales order in CRC cancer cell lines.

Genus	Isolated from	Extraction	Isolated Compounds	Cell Line/ /Administration Cytotoxicity Assay	Compound and IC_50_ or Cell Death (%)	Reference
*Alternaria*	*Miquelia dentata*	Methanol Ethyl acetate (EtOAc)	-	SW-480 and HCT-116 - Hoechst 33342	Methanol extract: HCT-116 (5.39 μg/mL), SW-480 (12.37 μg/mL); EAE: HCT-116 (6.59 μg/mL), SW-480 (7.2 μg/mL)	[77]
*Alternaria*	*Erythrophleum fordii*	Ethanol	(6a*R*, 6b*S*, 7*S*)-3, 6a, 7, 10-tetra-hydroxy-4, 9-dioxo-4, 6a, 6b, 7, 8, 9-hexahydroperylene (1)	HCT-8 DMSO MTT	(1): 1.78 μmol/L	[78]
*Bipolaris*	Soil	EtOAc	-	HCT-8 and HCT-116 DMSO CellTiter-Glo assay	Extract: HCT-8 (202.5 μg/mL), HCT-116 (18.97 μg/mL)	[67]
*Drechslera*	-	Ethanol	*di*-2-ethylhexyl phthalate	HCT-116 DMSO MTT	*Drechslera* extract: 104.0; *di*-2-ethylhexyl phthalate: 9.5	[23]
*Paradendryphiella*	*Pomacea canaliculata*	EtOAc	(*3R*, *6R*) hyalodendrin	SW-48, DLD-1, LS513, LOVO, RKO, LS174T, SW-480 HT-29 and HCT-116 DMSO MTT	(*3R*, *6R*) hyalodendrin: SW-48 (149.0 nM), DLD-1 (40.0 nM), HT-29 (58.0 nM), HT-29 5FU (146.8 nM), HT-29 oxa (141.8 nM), HT-29 SN-38 (93.8 nM), HCT-116 (48.0 nM), HCT-116 5FU (72.0 nM), HCT-116 oxa (25.7 nM), HCT-116 SN-38 (43.8 nM), LS513 (78.0 nM), LOVO (73.4 nM), RKO (74.3 nM), LS174T (158.0 nM), SW-480 (163.7 nM)	[80]
*Phoma* *Curvularia* *Pleosporales* sp. *Alternaria*	*Catharanthus roseus* *Euphorbia prostrata* *Calotropis procera*	EtOAc	-	HT-29 and HCT-116 - MTT	*C. aeria* extract: HT29 (74.5 μg/mL), HCT-116 (53.9 μg/mL); *Pleosporales* sp. extract: HT29 (69.4 μg/mL), HCT-116 (36.7 μg/mL); *P. multirostrata* extract: HT29 and HCT-116 (>100 μg/mL); *C. australiensis* extracts 1: HT29 (54.3 μg/mL), HCT-116 (25.6 μg/mL); 2: HT29 (>100 μg/mL), HCT-116 (59.7 μg/mL); *A. alternata* extract: HT29 (>100 μg/mL), HCT-116 (52.5 μg/mL); *Alternaria* sp. extract: HT29 (28.4 μg/mL), HCT-116 (29.1 μg/mL)	[39]
*Pleosporales* sp.	*Bacopa monnieri*	Dichloromethane	-	HCT-116 DMSO MTT	CK01: O (12 μg/mL), A (>100 μg/mL)	[66]
*Setophoma*	Leaf litter	Methanol/ Chloroform (1:1)	Secalonic acid A (1), E (2) and G (3) Penicillixanthone A (4) and B (5) Blennolide J (6) Hypothemycin (7)	SW-620 DMSO CellTiter 96 AQueous One Solution Cell Proliferation Assay	(1): 0.41 μM; (2): 19.12 μM; (3): 3.67 μM; (4): 0.21 μM); (5): 5.55 μM; (6): 6.14 μM; (7): 2.14 μM	[79]

HCT-116/HT-29 oxa: oxaliplatin resistant cell; HCT-116/HT-29 5FU: 5-fluorouracil resistant cell; HCT-116/HT-29 SN-38: SN-38 resistant cell; MTT: 3-(4,5-dimethytlthiazol-2-yl)-2,5-diphenyltetrazolium bromide; IC_50_: half maximal inhibitory concentration; DMSO: dimethyl sulfoxide; CK01: Pleosporales sp. extract; O: organic residue; A: water extract; SN-38: 7-Ethyl-10-hydroxycamptothecin; EAE: ethyl acetate extract.

**Table 4:** Antitumor activity of the extracts or isolated compounds from Sordariales order in CRC cancer cell lines.

Genus	Isolated from	Extraction	Isolated Compounds	Cell Line/ /Administration Cytotoxicity Assay	Compound and IC_50_ or Cell Death (%)	Mechanism of Action	Reference
*Chaetomium*	-	Liquid medium: Ethyl acetate (EtOAc) Mycelia: Methanol	Chaetocochin (Ch.) J	RKO, SW-480 and HCT-116 - CCK8	Ch. J: RKO (0.56 μM), SW-480 (0.61 μM), HCT-116 (0.65 μM)	Ch. J. induces apoptosis, autophagy and activates AMPK and PI3K/AKT/mTOR signaling pathway	[83]
*Chaetomium*	-	EtOAc	-	HCT-116 DMSO SRB	*C. globosum* extract: 1.2 μg/ml	-	[49]
*Chaetomium*	Soil	EtOAc	-	HCT-8 and HCT-116 DMSO CellTiter-Glo assay	Ethyl acetate extract (EAE): HCT-8 (8.744 μg/mL), HCT-116 (152.8 μg/mL)	-	[67]
*Chaetomium*	*Cymbidium goeringii*	Liquid medium: EtOAc Mycelia: Methanol	Ch. A and C	SW-480 Alone MTT	Ch. A: 15.21 μM Ch. C: 0.63 μM	Ch. C. induces G2/M arrest, apoptosis, activation of the caspase 3 and PARP degradation, increased Bax and decreased Bcl-2 level	[82]
*Chaetomium*	*Trigonella foenum-graecum*	EtOAc	-	HT-29 and HCT-116 - MTT	*C. globosum* extract: HT-29 (>100 μg/mL), HCT-116 (75.2 μg/mL)	-	[39]
*Chaetomium*	*Ginkgo biloba*	Methanol	Chaetoglobosin A (1), G (2), V (3), Vb (4), E (5), F (6), Fex (7), Fa (8) 20-dihydrochaetoglobosin A (9)	HCT-116 Alone SRB	(1): 3.15 μM; (2): 65.6 μM; (3): 29.5 μM; (4): 18.4 μM; (5): >100 μM; (6): 17.8 μM; (7): 17.8 μM; (8): 5.85 μM; (9): 8.44 μM	-	[81]
*Trichlocladium*	Soil	EtOAc	Trichocladinols E (1), F (2) and G (3)	SW-480 and HCT-116 DMSO MTS	(1): SW-480 (54.9 μM), HCT-116 (48.8 μM); (2): SW-480 (51.9 μM), HCT-116 (56.6 μM); (3): SW-480 (43.6 μM), HCT-116 (41.7 μM)	-	[84]
*Scytalidium*	-	Methanol/Chloroform (1:1)	[5’-formyl-2’-hydroxyl-4’-methoxy-(*E,E*)-sorbophenone (1) Scalbucillin B (2) 1-(2’-hydroxy-4’-methoxy-5’-methylphenyl)-2,4-*E,E*-hexadien-1-one (3) 5′-formyl-2′-hydroxy-4′-methoxy-(*E*)-4-hexenophenone (4)	SW-620 DMSO CellTiter 96^®^ AQueous One Solution Cell Proliferation Assay	(1): 0.5 μM; (2): 16 μM; (3): 15.1 μM; (4): 2.5 μM	-	[85]

SRB: sulforhodamine B; CCK8: cell counting kit-8; MTS: ((3-(4,5-dimethylthiazol-2-yl)-5-(3-carboxymethoxyphenyl)-2-(4-sulfophenyl)-2H-tetrazolium)) assay; MTT: 3-(4,5-dimethytlthiazol-2-yl)-2,5-diphenyltetrazolium bromide; IC_50_: half maximal inhibitory concentration; DMSO: dimethyl sulfoxide; AMPK: AMP-activated protein kinase; PARP: poly (ADP-ribose) polymerase.

## Data Availability

Data sharing not applicable.

## Supplementary Materials

The following supporting information can be downloaded at: https://www.mdpi.com/article/10.3390/ph16010022/s1.

## Abbreviations

CRC	Colorectal cancer
EGFR	Epidermal growth factor receptor
VEGF	Vascular endothelial growth factor
DMSO	Dimethyl sulfoxide
MTT	3-(4,5-dimethytlthiazol-2-yl)-2,5-diphenyltetrazolium bromide
Asp. A	Asperphenins A
Asp. B	Asperphenins B
SRB	Sulforhodamine B
ROS	Reactive oxygen species
TXL	Paclitaxel
EtOAc	Ethyl acetate
CH_2_Cl_2_ extract	Sequential ethyl acetate, methanol, and dichloromethane extract
Dox	Doxorubicin
CCK8	Cell counting kit-8
WST-1	4-[3-(4-Iodo-phenyl)-2-(4-nitrophenyl)-2H-5 tetrazolio]-1,3-benzene disulphonate
RTCA-DP	Real-Time Cell Analysis System
EAE	Ethyl acetate extract
MTS	((3-(4,5-dimethylthiazol-2-yl)-5-(3-carboxymethoxyphenyl)-2-(4-sulfophenyl)-2H-tetrazolium)) assay
Mrna	Messenger RNA
IC_50_	Half maximal inhibitory concentration
GSC	*Cordyceps militaris* cultivated on germinated soybeans
EE	Ethanol extract
HCT-116/HT-29 oxa	Oxaliplatin resistant cell
HCT-116/HT-29 5FU	5-fluorouracil resistant cell
HCT-116/HT-29 SN-38	SN-38 resistant cell
CK01	*Pleosporales* sp. Extract
SN-38	7-Ethyl-10-hydroxycamptothecin
AMPK	AMP-activated protein kinase
PARP	Poly (ADP-ribose) polymerase
PMEP	Polysaccharides extracted from *Morchella angusticepes* Peck
Ac-PMEP	Acetylated derivatives of PMEP
AChE	Acetylcholinesterase
PLEP	Phosphorylated polysaccharide
SLEP	Sulfated polysaccharide
