# Supplementary material for: In Vitro Efficacy of Extracts and Isolated Bioactive Compounds from Ascomycota Fungi in the Treatment of Colorectal Cancer: A Systematic Review
Source: Pharmaceuticals (Basel). 2022 Dec 23;16(1):22. doi: 10.3390/ph16010022 (PMC9864996; doi:10.3390/ph16010022)
Supplement: Supplementary file 1 [file pharmaceuticals-16-00022-s001.zip › pharmaceuticals-2070296-supplementary materials.pdf]

SUPPLEMENTARY MATERIAL

In Vitro Efficacy Of Extracts And Isolated Bioactive Compounds From Ascomycota Fungi In The Treatment Of Colorectal Cancer: A Systematic Review

Table S1. Bioactive compounds isolated from different orders from Ascomycota.

**Bioactive compound**	**Structures of compounds**	**Isolated from Order**	**Referenc e**
Asperphenins A (1)<br>Asperphenins B (2)	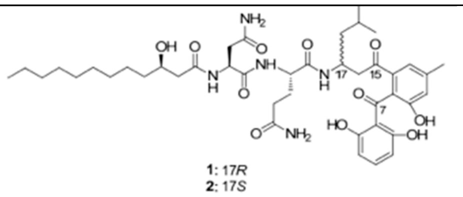	Eurotiales	[20]
Allianthrone A (4), B (5) and C (6)	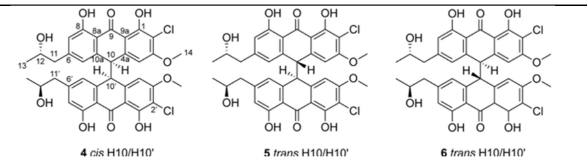	Eurotiales	[34]
Aspergiside B (2)<br>Aspergisidone (4)<br>Emeguisin A (5)<br>Folipastatin (6)<br>Aspergillusidone C (9)<br>Unguinol (10)<br>2-Chlorounguinol (11)<br>2,4-Dichlorounguinol (14)<br>Nidulin (15)	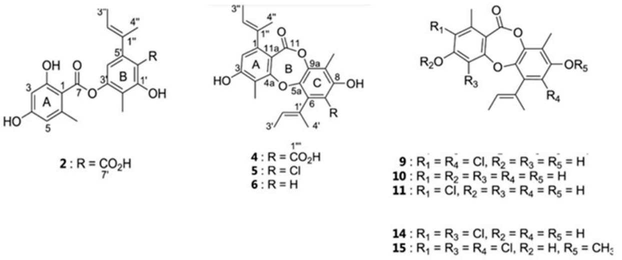	Eurotiales	[30]
Violaceimide A-E (1-5)	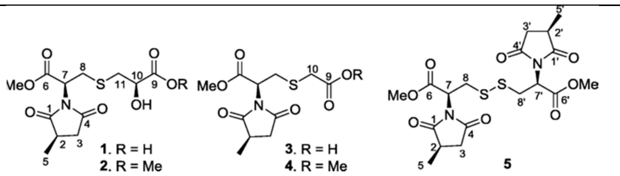	Eurotiales	[35]
Malformin C	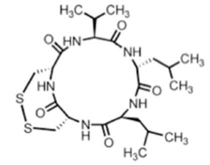	Eurotiales	[40]
Clavatustide B	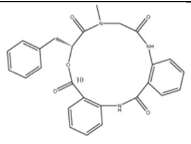	Eurotiales	[28]
Ergosterol (1)<br>Rosellichalasin (2)<br>Cytochalasin E (3)	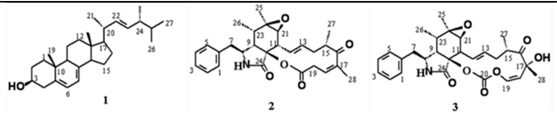	Eurotiales	[27]

Asperterone B (1) and C (2)	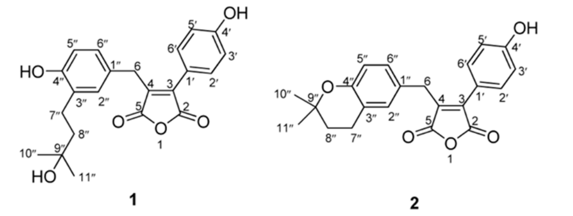	Eurotiales	[41]
Isocoumarin (R)-mellein (1)<br>Penicillic acid (2)<br>*cis*-4-hydroxymellein (3)<br>*trans*-4-hydroxymellein (4)	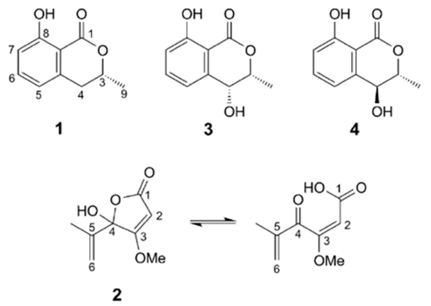	Eurotiales	[26]
Acetylaranotin (1)<br>Acetylapoaranotin (2)<br>Deoxyapoaranotin (3)	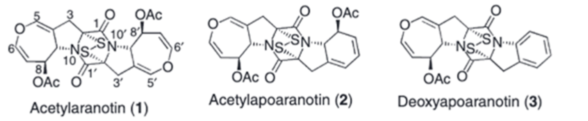	Eurotiales	[42]
Pityriacitrin	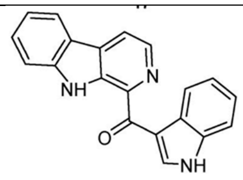	Eurotiales	[92]
Bis(dethio)bis(methylsulfanyl)gliotoxin (1)<br>6-acetylbis(dethio)bis(methylsulfanyl)gliotoxin (2)<br>Acetylgliotoxin G (3)<br>Gliotoxin (4)<br>Acetylgliotoxin (5)<br>Fiscalin B (6)	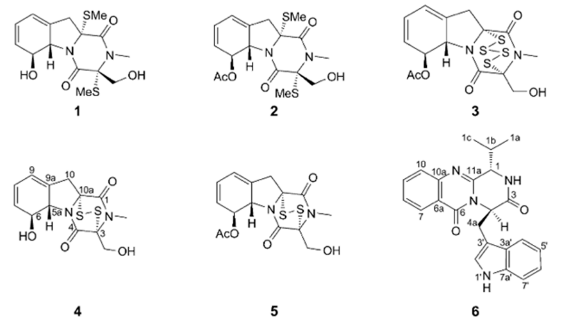	Eurotiales	[44]
1,8-Dihydroxy-3-methoxy-6-methyl-anthraquinone	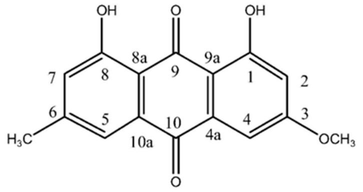	Eurotiales	[23]
Chevalone C (2)<br>Nortryptoquivaline (4)<br>Tryptoquivaline H (6)<br>Fiscalin A (8)<br>*epi*-fiscalin A (9) and C (13)<br>*epi*-neofiscalin A (11)	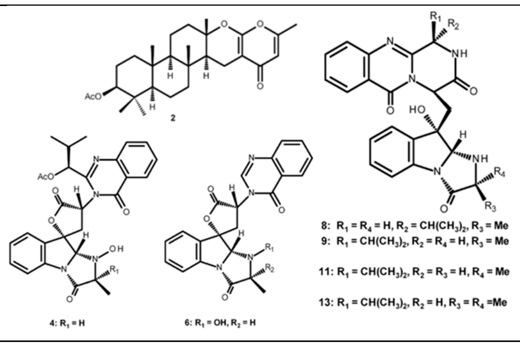	Eurotiales	[46]

1,2,3,4-Tetrahydro-2-methyl-3-methylene-1,4-dioxopyrazino[1,2-a]índole (4)<br>1,2,3,4-Tetrahydro-2-methyl-1,3,4-trioxopyrazino[1,2-a]índole (5)<br>Reduced gliotoxin (9)<br>6-Acetylbis(methylthio)gliotoxin (10)<br>Bisdethiobis(methylthio)gliotoxin (11)<br>Didehydrobisdethiobis(methylthio)gliotoxin (12)<br>Bis-*N*-norgliovictin (13)	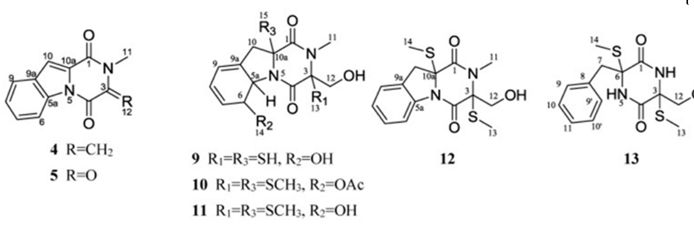	Eurotiales	[48]
Isopenicin A	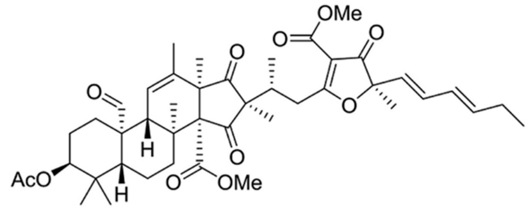	Eurotiales	[50]
Arenicolin A	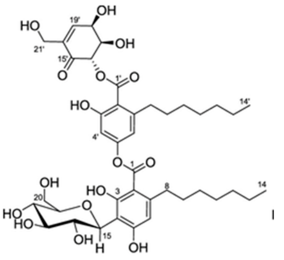	Eurotiales	[21]
cis-bis(methylthio)silvatin	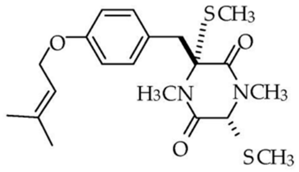	Eurotiales	[51]
Epoxydon (1)<br>3,6,8-trihydroxy-1-methylxanthone (2)<br>Gentisyl alcohol (3)<br>(*R,S*)-1-phenyl-1,2-ethanediol (4)<br>Dehydrodechlorogriseofulvin (5)<br>Dechlorogriseofulvin (6)<br>Griseofulvin (7)<br>Ethylene glycol benzoate (8)<br>Alternariol (9)<br>Griseoxanthone C (10)<br>Drimiopsin H (11)<br>Griseophenone B (12) and C (13)	Not specified	Eurotiales	[53]
Kongiiline A (1), B (7)<br>Pebrolide (2)<br>1-deoxypebrolide (3)<br>Asperphenamate (4)<br>Asperphenamate B (5), C (6)<br>N-benzoyl-phenylalaninol (8)<br>Orsellinic acid (9)<br>Mycophenolic acid (10)<br>5, 7-dihydroxy-4-methylphthalide (11)	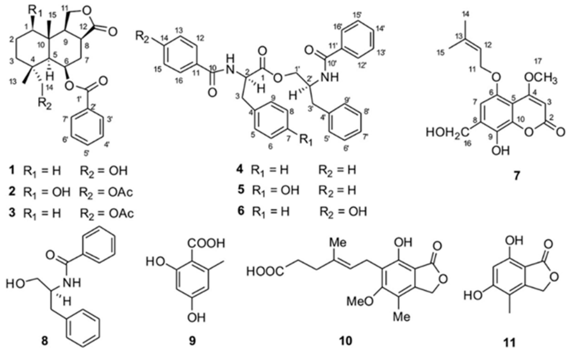	Eurotiales	[55]

(–)-Brevianamide C	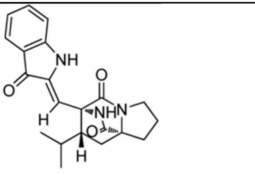	Eurotiales	[29]
Fructigenine A (3)<br>Verrucosidin (8)<br>Norverrucosidin (9)	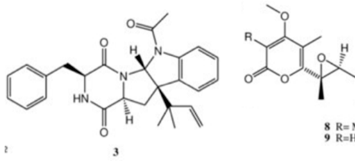	Eurotiales	[30]
Penipacids A (1) and E (5)	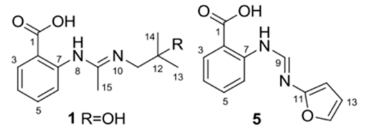	Eurotiales	[57]
1-Hydroxy-10-methoxy-dibenz[b, e]oxepin-6,11-dione (1)<br>Chrysazin (2)<br>Globosuxanthone A (3)	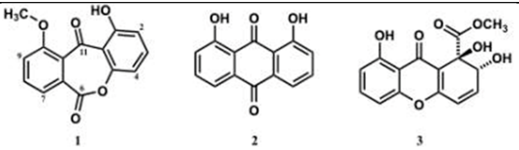	Hypocreales	[73]
Camptothecin	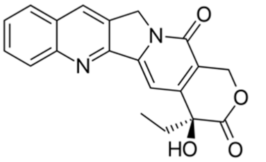	Hypocreales	[69]
Destruxin A, B and E	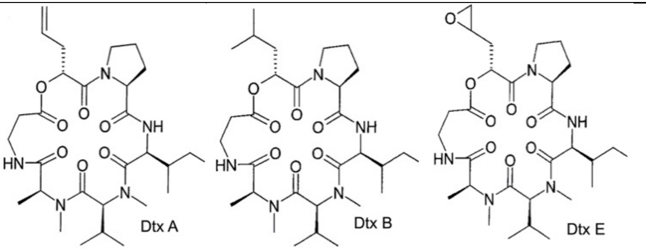	Hypocreales	[75]
Trichodermaloid A (1), B (2) and C (5)<br>Aspergilloid G (3)<br>Rhinomilisin E (4) and G (6)	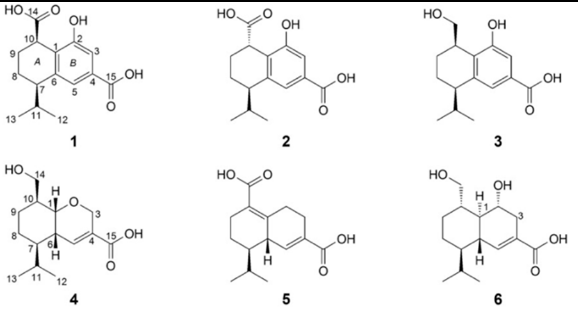	Hypocreales	[72]
(6a*R*, 6b*S*, 7*S*)-3, 6a, 7, 10-tetra-hydroxy-4, 9-dioxo-4, 6a, 6b, 7, 8, 9-hexahydroperylene	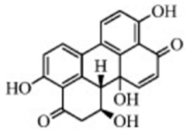	Pleosporales	[79]
di-2-ethylhexyl phthalate	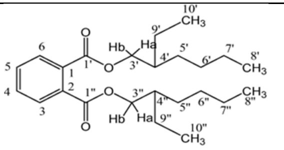	Pleosporales	[23]
(3*R*, 6*R*) hyalodendrin	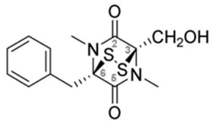	Pleosporales	[81]

Secalonic acid A (1), E (2) and G (3)<br>Penicillixanthone A (4) and B (5)<br>Blennolide J (10)<br>Hypothemycin (12)	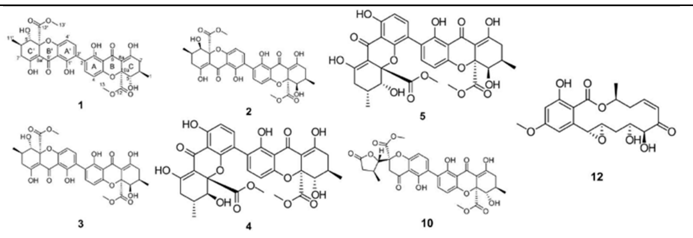	Pleosporales	[80]
Chaetocochin J	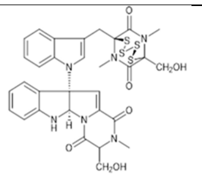	Sordariales	[84]
Chaetocochin A (2) and C (3)	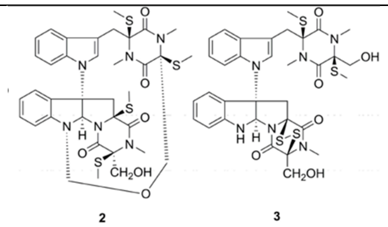	Sordariales	[83]
Chaetoglobosin A (1), G (2), V (3), Vb (4), E (6), F (7), Fex (8), Fa (10)<br>20-dihydrochaetoglobosin A (9)	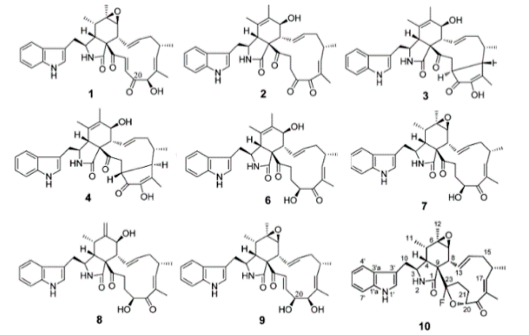	Sordariales	[82]
Trichocladinols E (2), F (3) and G (4)	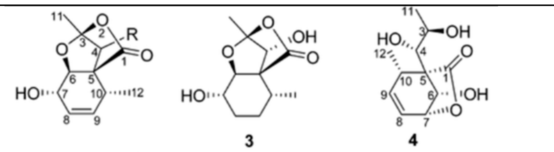	Sordariales	[85]
[5'-formyl-2'-hydroxyl-4'-methoxy-(*E*,*E*)-sorbophenone (1)<br>Scalbucillin B (3)<br>1-(2'-hydroxy-4'-methoxy-5'-methylphenyl)-2,4-*E*,*E*-hexadien-1-one (4)<br>5′-formyl-2′-hydroxy-4′-methoxy-(*E*)-4-hexenophenone (5)	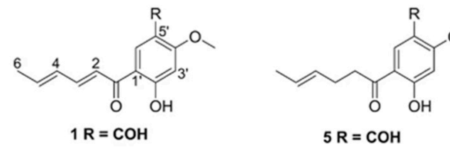	Sordariales	[86]
8,8′-Bijuglone	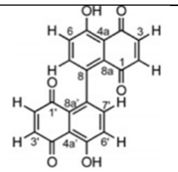	Capnodiales	[88]
Taxol	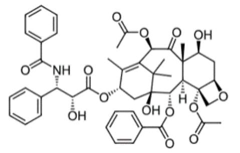	Capnodiales	[87]

Phomolactonexanthone A (1), B (2) and C (3)<br>Dicerandrol A (4), B (5) and C (6)<br>Deacetylphomoxanthone B (7)<br>Penexanthone A (8)	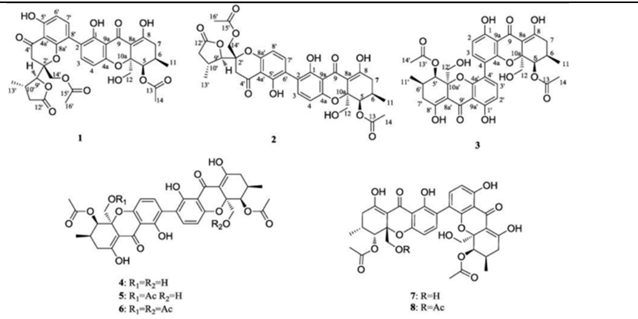	Diaporthales	[93]
5-methylmellein	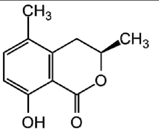	Xylariales	[96]
Daldinone F (1)<br>Nodulisporin G (2)<br>Dalmanol C (3)	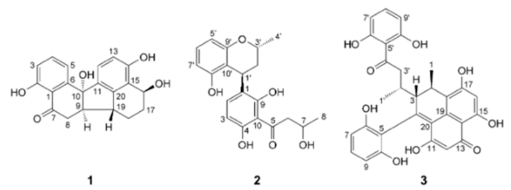	Xylariales	[97]
Xylarenone C (6) and D (7)	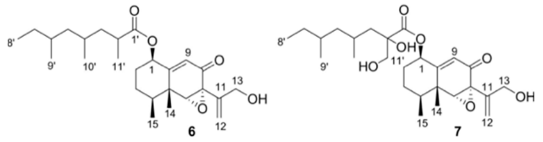	Boliniales	[99]
Greensporone A (1), C (5)<br>Dechlorogreensporone A (4), D (10)<br>*O*-Desmethylgreensporone C (6)	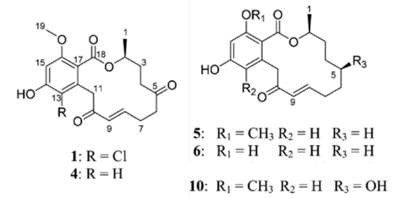	Leotiales	[98]
